# Perception of sexuality and fertility in women living with HIV: a questionnaire study from two Nordic countries

**DOI:** 10.7448/IAS.18.1.19962

**Published:** 2015-06-01

**Authors:** Maria Wessman, Inka Aho, Kristina Thorsteinsson, Merete Storgaard, Isik S Johansen, Suzanne Lunding, Gitte Pedersen, Anne-Mette Lebech, Pia Kivelä, Marie Helleberg, Terese L Katzenstein, Nina Weis

**Affiliations:** 1 Department of Infectious Diseases, Copenhagen University Hospital Hvidovre, Copenhagen, Denmark; 2 Department of Infectious Diseases, Helsinki University Central Hospital, Helsinki, Finland; 3 Department of Infectious Diseases, Aarhus University Hospital, Aarhus, Denmark; 4 Department of Infectious Diseases, Odense University Hospital, Odense, Denmark; 5 Department of Pulmonary Medicine and Infectious Diseases, North Zealand Hospital, Hillerød, Denmark; 6 Department of InfectiousDiseases, Aalborg University Hospital, Aalborg, Denmark; 7 Department of Infectious Diseases, Copenhagen University Hospital, Rigshospitalet, Copenhagen, Denmark; 8 Department of Clinical Medicine, Faculty of Health and Medical Sciences, University of Copenhagen, Copenhagen, Denmark

**Keywords:** HIV, women, questionnaire, sexuality, fertility

## Abstract

**Introduction:**

As the human immunodeficiency virus (HIV)-positive population ages, issues concerning sexuality and fertility, among others, are becoming relevant. HIV is still surrounded by stigma and taboos, and there have been few studies conducted in industrialized settings concerning these questions. We therefore wanted to investigate the perception of sexuality and fertility in women living with HIV (WLWH) in an industrialized setting, using a questionnaire.

**Methods:**

WLWH were recruited at their regular outpatient clinic visits, at the major Departments of Infectious Diseases in Denmark and Finland, from January 2012 to October 2013. A questionnaire was developed, study participants were informed of the nature of study and, if they agreed to participate and signed a consent form, they filled in the questionnaire. Demographic information on the participants was obtained from patient files (in Finland) or from a national HIV cohort (in Denmark). Statistical analysis was performed using STATA^®^, version 11.

**Results:**

In total, 560 women were included in the study. The median age was 44 years. The majority were of white European origin, with fully suppressed HIV viral load, CD4 cell count >350 µL and mild or no symptoms of their HIV infection. A total of 62% were sexually active, stating condom use as their sole form of contraception. Of the sexually inactive women, one-third were in steady relationships. Eighty percent reported prior pregnancies, of which the majority had one or more children. Most children were born prior to the women's HIV diagnosis and the mode of conception was predominantly natural. One-quarter of the participating women desired pregnancy, while more than half did not. The remaining quarter either stated that they already had the desired number of children or chose not to answer the question. Fourteen percent stated that their HIV diagnosis ended their wish for children; of these women, the median time of diagnosis was between 1995 and 1996. Pregnancy had been attempted unsuccessfully in one-quarter of study participants. The final question inquired what the risk of mother-to-child transmission was, with all precautions taken. Fifteen percent estimated the risk to be above two percent.

**Conclusions:**

In conclusion, the majority of WLWH in industrialized settings in Denmark and Finland have few HIV-related symptoms, are sexually active and have a strong desire for children.

## Introduction

In 2013, 35 million people were living with human immunodeficiency virus-1 (HIV) [1], half of them women [2]. Since 1995, it has been possible to offer people living with HIV (PLWH) effective antiretroviral treatment (ART). PLWH therefore have a median life expectancy approaching that of the HIV-negative population [3,4]. With prolonged longevity, the HIV-positive population is ageing and issues concerning sexuality and fertility have become relevant. Despite the improved treatment and quality of life, HIV is still surrounded by stigma and taboos. PLWH often feel stigmatized and isolate themselves with their illness [5,6]. In a cohort of 340 heterosexual HIV-positive individuals included in a Danish survey, 11% felt that others were anxious and kept a physical distance and 25% had at some point felt isolated [7]. Additionally, almost 50% stated that their HIV diagnosis altered their sex life significantly. One-quarter reported a need for more information concerning sexual problems, and 30% wanted health personnel at outpatient clinics to take the initiative to discuss sexual issues [7].

A 2013 systematic review stressed the urgent need for access to family planning counselling and HIV prevention services in low- and middle-income countries; it highlighted the high demand for studies concerning fertility intentions and desires among HIV-positive women [8]. Surveying the past 21 years, only nine relevant studies, all conducted in Africa, were found. The authors of the review concluded that there are few studies and little knowledge concerning the fertility and desire for pregnancy of women living with HIV (WLWH), as well as the optimal use of contraceptives [8]. This large gap needs to be filled in order to provide health personnel and policy makers with the proper knowledge, so that targeted interventions can be offered to WLWH and their partners [8].

Most studies concerning fertility among PLWH are from developing countries. The few existing data regarding the female HIV-positive population indicate that WLWH have lower fertility rates than HIV-negative women [9–11].

Therefore, the aim of the present study was to elucidate the perception of sexuality and fertility among WLWH, in an industrialized setting with free access to treatment and care.

## Methods

Study participants were recruited from all major outpatient clinics at the Departments of Infectious Diseases in Denmark and the largest Finnish outpatient clinic at the Department of Infectious Diseases, Helsinki University Hospital, from January 2012 to October 2013.

Women were recruited at their regular outpatient clinical visits and, if agreeing to participate, had the opportunity to fill in the questionnaire either before or after the planned appointment. The estimated time for questionnaire completion was 15 to 20 minutes (Supplementary Appendix). Most women that consented to participate in the study filled in the questionnaire in immediate association with their outpatient visit. The women that wished to consider their participation in the study had the opportunity to either fill in the questionnaire at their next regular outpatient visit or choose to deliver the questionnaire at the Department when suitable.

### Inclusion and demographic data

Not all female patients at the clinics were asked to participate, either because of missed appointments or missed opportunities by health personnel. The number of women declining participation at the Danish recruiting sites was registered. Due to an unsuitable registration system, this information was not available in Finland.

Inclusion criteria were as follows: HIV positive, age >18 years and able to read and understand Danish, English, Finnish or Swedish. In Finland, a professional translator was available on site, to translate for the women not fluent in any of these four languages. Only five women used a translator, all from Asian countries (Thailand and Myanmar).

In Denmark, demographic data for the women included in the study was extracted from the Danish HIV Cohort Study, which is a prospective, population-based cohort initiated in 1998 with the purpose of conducting scientific studies of HIV in Denmark [12]. In Finland, this information was obtained from patient files.

### Questionnaire development

A prototype questionnaire was developed after research of the construction and composition of survey studies related to issues included in the present study [13–15]. In order to validate the questionnaire, a pilot study was performed with 15 women and the questions were adjusted where needed.

English was included due to the large number of HIV-positive non-native Danish/Finnish women living in both countries [12].

Of the questionnaire's 40 questions (Supplementary Appendix), we focused on the 33 questions concerning demographics, sexuality and fertility. Due to a large number of young premenopausal women answering questions on menopause, this part of the study was excluded.

The questionnaire was divided into segments. The first part included questions on demographic data; these were questions on marital status, self-perceived symptoms of HIV, smoking habits and education.

The second part of the questionnaire was about sexuality; these questions concerned sexual activity, use of contraception, type of contraception and, if no use was stated, the reasons for this were clarified.

The third part focused on fertility and the questions were about pregnancy: whether there were any prior pregnancies, mode of conception, any prior abortions or unsuccessful attempts, fertility examinations, desire for pregnancy, changes of views on having children after HIV diagnosis and possible influences of ART on desire for children.

The last part of the questionnaire focused on questions concerning menopause: whether the women had regular periods, reasons for and age of entering menopause, hormonal treatment and symptoms of menopause.

Finally, in order to obtain an impression of the information level of WLWH, the women were asked to estimate the risk of mother-to-child transmission (MTCT), all precautions taken.

For all questions, one of the response options was “Do not wish to answer.”

### Statistical analysis

Data were analyzed using STATA^®^, version 11 (Stata Corporation, College Station, TX, USA). Wilcoxon rank-sum test and Pearson chi-squared test were used in analyses comparing the demographic characteristics of the women participating and the women not participating in the study. Multivariate logistic regression was used to assess associations between demographic characteristics and sexuality and fertility.

### Ethics

Ethics approval is not required by Danish and Finnish legislation governing questionnaire studies [16,17]. The Danish Data Protection Agency approved the study. In Finland, approval from the Finnish Hospital Board was obtained.

## Results

A total of 1365 HIV-positive women were followed at the seven participating sites. The clinics care for >90% of the total HIV-positive female population in Denmark [12] and >50% of the total HIV-positive female population in Finland (IA, personal communication).

In total, 577 (42%) women were asked to participate in the study, of which 17 declined (all in Denmark) and 560 agreed to participate.

Unless further specified, the response option “do not wish to answer” and no response (area left blank) were both reported as “not answered.”

### Demographic characteristics

The median age at inclusion was 44 years (interquartile range [IQR] 25%, 37 years; IQR 75%, 50 years) and median age at HIV diagnosis was 31 years (IQR 25%, 26 years; IQR 75%, 38 years). The median age of the women not participating was 41.6 years (IQR 25%, 35 years, IQR 75%, 48 years). There was a significant difference in age distribution between the two groups. The majority of women were white, of European descent. Among the women of African origin (the largest ethnic group other than European), the vast majority were immigrants (144 of 178 women; for 31 women there was no information regarding whether they were immigrants or native to one of the two countries), who entered Denmark or Finland after the year 1990. There were significantly (*p*<0.05) fewer white European women among those not included in the study. At time of inclusion, the median CD4 cell count was 606 µL and 91% had HIV RNA ≤50 copies/ml, while on treatment. There were significantly (*p*<005) more women included in the study with a fully suppressed viral load and a high CD4 count than among those not included. Most of the women (*n*=478; 85%) had mild or no self-perceived symptoms of HIV, and 56 (10%) had moderate to severe symptoms. There was no significant difference between the number of women with previous AIDS diagnosis among the women participating and not participating.

Most women (*n*=363; 65%), were in a steady relationship; 188 (34%) were either divorced, widowed or single.

All demographic characteristics are presented in Table 1.

**Table 1:** Demographic and individual characteristics of participants

	Participants, *n* (%)
Total number of women	560
Age	
18 to 25	17 (3)
26 to 35	90 (16)
36 to 45	206 (37)
46 to 55	163 (29)
≥56	72 (13)
Age unknown	12 (2)
Ethnicity	
European	326 (58)
African	178 (32)
Asian	52 (9)
Unknown origin	4 (1)
Number of children	
0	90 (16)
1	148 (26)
2	194 (35)
≥3	87 (16)
Unknown number	41 (7)
Mode of transmission	
Heterosexual	469 (84)
IDU	41 (7)
Mother-to-child transmission	5 (1)
Blood/organ donation	8 (1)
Unknown	37 (7)
Current ART use	501 (89)
Prior ART use	5 (1)
CD4 cell count >350 µL	472 (84)
CD4 cell count 200 to 350 µL	50 (9)
CD4 cell count (×10^6^/l) <200	17 (3)
Unknown CD4 cell count	21 (4)
RNA viral ≤50 copies/mL (while on ART)	454 (91)
RNA viral >50 copies/mL	77 (14)
Unknown RNA viral load	21 (4)
Previous AIDS diagnosis	98 (18)
Symptoms of HIV	
Total	560 (100)
None	380 (68)
Mild	98 (18)
Moderate	36 (6)
Severe	20 (3)
NA	26 (5)
Smoking habit	
Total	560 (100)
Yes	154 (27)
Ex-smoker	95 (17)
No	295 (53)
NA	16 (3)
Number of cigarettes daily	
Total	249 (100)
<5	30 (12)
5 to 20	160 (64)
>20	22 (9)
NA	37 (15)
Smoking duration	
Total	249 (100)
<5 years	20 (8)
>5 years	203 (82)
NA	26 (10)
Education	
Total	560 (100)
Elementary	220 (39)
Grammar school or above	307 (55)
NA	33 (6)
Civil status	
Total	560 (100)
Married	215 (38.5)
Cohabiting	69 (12)
Steady relationship	79 (14)
Divorced	52 (9)
Widowed	17 (3)
Single	119 (21.5)
NA	9 (2)
Partner's HIV status	
Total	363 (100)
HIV positive	124 (34)
HIV negative	215 (59)
Unknown	21 (6)
NA	3 (1)

IDU, intravenous drug use; ART, antiretroviral therapy; NA, not answered.

### Sexuality

A total of 344 (62%) women were sexually active and 178 (32%) were not. Of the sexually active women, 169 (49%) reported having had sex within the last week, 101 (29%) within the last month, 58 (17%) within the last six months and 16 (5%) gave no answer.

Of the sexually inactive women, 57 (32%) were in a steady relationship, and 117 (66%) were not. The median age of the 57 sexually inactive women in a steady relationship was 44 years, and the median age of the 283 sexually active women in a steady relationship was 42 years. A significant number of women in a steady relationship were sexually active, compared to the women not in a steady relationship (Table 2).

**Table 2:** Multivariate logistic regression analysis of demographics, sexual activity, contraception, pregnancy desires and - attempts and perceived risk of MTCT among WLWH

	Sexually active	No contraception	Desire for pregnancy	Pregnancy attempt without success	Perceived risk of MTCT
Outcome	OR (95% CI)	OR (95% CI)	OR (95% CI)	OR (95% CI)	OR (95% CI)
Age<35 years	1.0 (ref.)	1.0 (ref.)	1.0 (ref.)	1.0 (ref.)	1.0 (ref.)
Age≥35 years	0.65 (0.35 to 1.18)	1.53 (0.79 to 2.98)	**0.53 (0.30 to 0.94)**	1.82 (1.00 to 3.32)	**2.32 (1.07 to 5.05)**
CD4 count					
>350	1.0 (ref.)	1.0 (ref.)	1.0 (ref.)	1.0 (ref.)	1.0 (ref.)
200 to 349	0.96 (0.44 to 2.09)	**3.31 (1.37 to 8.00)**	**2.85 (1.18 to 6.89)**	**0.74 (1.36 to 5.55)**	0.94 (0.37 to 2.38)
<200	0.50 (0.15 to 1.72)	1.80 (0.33 to 9.91)	3.36 (0.53 to 21.14)	0.77 (0.20 to 2.97)	0.85 (0.17 to 4.28)
HIV RNA					
<50	1.0 (ref.)	1.0 (ref.)	1.0 (ref.)	1.0 (ref.)	1.0 (ref.)
>50	0.74 (0.39 to 1.41)	0.63 (0.27 to 1.47)	0.60 (0.28 to 1.32)	**0.31 (0.14 to 0.72)**	1.24 (0.58 to 2.64)
Ethnicity					
European	1.0 (ref.)	1.0 (ref.)	1.0 (ref.)	1.0 (ref.)	1.0 (ref.)
Asian	0.58 (0.27 to 1.25)	1.13 (0.46 to 2.74)	0.86 (0.38 to 1.96)	0.41 (0.17 to 1.02)	0.79 (0.30 to 2.02)
African	1.32 (0.80 to 2.17)	1.03 (0.57 to 1.84)	1.46 (0.80 to 2.67)	**1.62 (1.02 to 2.58)**	1.19 (0.67 to 2.12)
Relationship status					
Single	1.0 (ref.)	1.0 (ref.)	1.0 (ref.)	1.0 (ref.)	1.0 (ref.)
Steady relationship	**5.05 (1.49 to 17.12)**	0.94 (0.15 to 5.77)	2.25 (0.39 to 12.94)	1.75 (0.39 to 7.81)	2.13 (0.31 to 14.88)
Education					
Elementary school	1.0 (ref.)	1.0 (ref.)	1.0 (ref.)	1.0 (ref.)	1.0 (ref.)
Grammar school or higher	0.96 (0.61 to 1.53)	1.04 (0.62 to 1.77)	1.21 (0.68 to 2.17)	0.82 (0.52 to 1.28)	**0.35 (0.21 to 0.60)**
Children					
No	1.0 (ref.)	1.0 (ref.)	1.0 (ref.)	1.0 (ref.)	1.0 (ref.)
Yes	1.13 (0.68 to 1.88)	0.65 (0.36 to 1.19)	0.73 (0.40 to 1.32)	0.94 (0.56 to 1.57)	1.02 (0.55 to 1.88)
Partner's HIV status					
Unknown	1.0 (ref.)	1.0 (ref.)	1.0 (ref.)	1.0 (ref.)	1.0 (ref.)
HIV positive	1.68 (0.57 to 4.96)	3.77 (0.94 to 15.22)	1.72 (0.33 to 8.92)	**0.34 (0.12 to 0.99)**	1.01 (0.24 to 4.25)
HIV negative	2.56 (0.88 to 7.43)	1.35 (0.34 to 5.34)	1.57 (0.32 to 7.82)	**0.32 (0.11 to 0.89)**	0.63 (0.15 to 2.60)

MTCT, mother-to-child transmission; ref., reference. Significant results are in bold print.

Of the participants, 304 (54%) women used contraceptives, 208 (37%) did not use contraceptives and 48 (9%) gave no answer. Of the sexually active women, 238 (70%) used contraception. The majority reported condoms as their sole mode of contraception, and the remaining women reported evenly distributed use of contraceptives between hormonal contraception, intrauterine devices and sterilization, all with or without condom use (Table 3).

**Table 3:** Contraceptive use

Use of contraceptives	Participants, *n* (%)
Total	304 (100)
Condom	206 (68)
Condom+IUD	22 (7)
Condom+hormonal	20 (6.5)
Condom+sterilization	15 (5)
Hormonal	12 (4)
IUD	13 (4)
Sterilization	11 (4)
Other	4 (1)
NA	1 (0.5)
Reason for not using contraception	
Total	208 (100)
Sexually inactive	74 (36)
Joint decision between partners	34 (16)
Partner also HIV positive	19 (9)
Own decision	17 (8)
Attempting pregnancy	12 (6)
Low viral load	13 (6)
Partner's decision	10 (5)
NA	29 (14)

IUD, intrauterine device; hormonal, hormonal contraception, including contraceptive pills and implants; NA, not answered. Modes of contraception for the 304 women who reported using contraceptives, and reasons for not using contraception for the 208 women who reported not using contraceptives.

The reasons for not using contraceptives are presented in Table 3.

### Fertility

A majority, 448 (80%) women, reported prior pregnancies. Twenty-six (4%) women were pregnant at the time of study, 70 (13%) had never been pregnant and 16 (3%) gave no answer.

Of the women who reported previous or current pregnancies, 393 (83%) had children and 78 (16%) had no children. The remaining 1% gave no answer. The women had a total of 685 children (one adopted), with a median age of 19 years.

A total of 21 (5%) women reported having HIV-infected children; 17 (81%) had one and four (19%) had two. Of the 25 HIV-infected children, 22 (88%) were born prior to the women's HIV diagnoses, two (8%) after and one (4%) gave no answer. For one of these children, born prior to the mother's HIV diagnosis, MTCT did not occur. Of the women who reported previous or current pregnancies, 49 (10%) had had an induced abortion *after* their HIV diagnosis, 39 (8%) had had a miscarriage, 70 (15%) a live birth, 4 (1%) a stillbirth, 4 (1%) had had two or more of the aforementioned events, 236 women (50%) had had none of the above and 72 (15%) gave no answer.

Out of 474 women who reported current or previous pregnancies, 111 (23%) were pregnant at the time of HIV diagnosis. Of those pregnancies, 76 (69%) resulted in a live birth, 18 (16%) in an induced abortion, 7 (6%) in a miscarriage, 1 (1%) in a stillbirth and 9 (8%) gave no answer.

**Figure 1:**
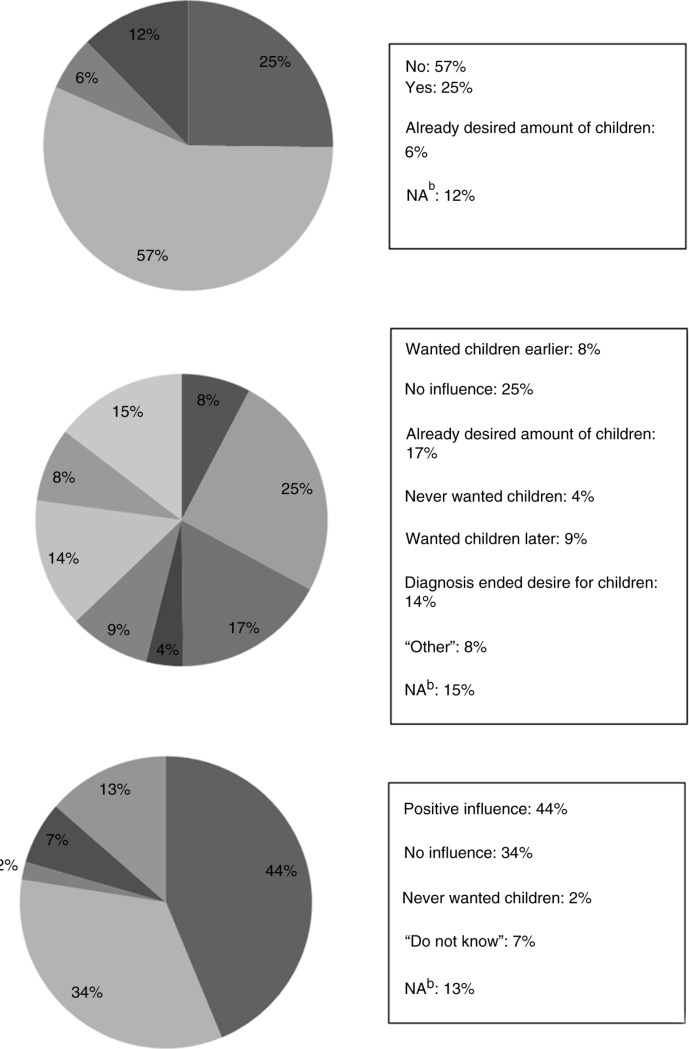
Desire for pregnancy and influence of HIV diagnosis and treatment on desire for children. Participants were asked the following questions: (a) Do you wish to become pregnant? (b) Has your HIV diagnosis changed your view on having children? (c) Have the improved treatment possibilities influenced your desire to have children?

Pregnancy was desired by 141 (25%) women and not desired by 316 (57%) women (Figure 1).

The median age of the women who desired pregnancy was 37 years, and the median age of the women not wanting pregnancy was 47 years. Women below the age of 35 had a significantly higher desire for children than women between the ages of 35 to 45 years (Table 2). Also, women with CD4 counts between 200 and 350 had a significantly larger desire for children than women with CD4 counts >350 (Table 2).

For 80 (14%) women, the HIV diagnosis ended their desire for children. The median time of HIV diagnosis for these women was between 1995 and 1996 (time of diagnosis was lacking for six women). The influence of HIV diagnosis on desire for children is presented in Figure 1. The improved treatment possibilities influenced 245 (44%) of the women in a positive way, concerning their desire for children (Figure 1).

A quarter of the women (134) had attempted pregnancy unsuccessfully. Of the 56 (10%) women attempting pregnancy at the time of the study, 9 (16%) had been trying for less than six months, 9 (16%) between 6 and 18 months and 28 (50%) for more than 18 months. The remaining 10 (18%) women gave no answer.

Of the women attempting pregnancy for more than 18 months, only 15 (54%) had undergone a fertility examination. Women of African origin had attempted pregnancies without success more often than women of Asian or European origin (Table 2). The probability of not becoming pregnant was lower if the partner was HIV positive and higher if the CD4 count of the woman was <350 (Table 2).

Forty-six women (8%) had been sterilized after being diagnosed with HIV, of whom 14 (30%) regretted the decision.

### Mother-to-child transmission

**Figure 2:**
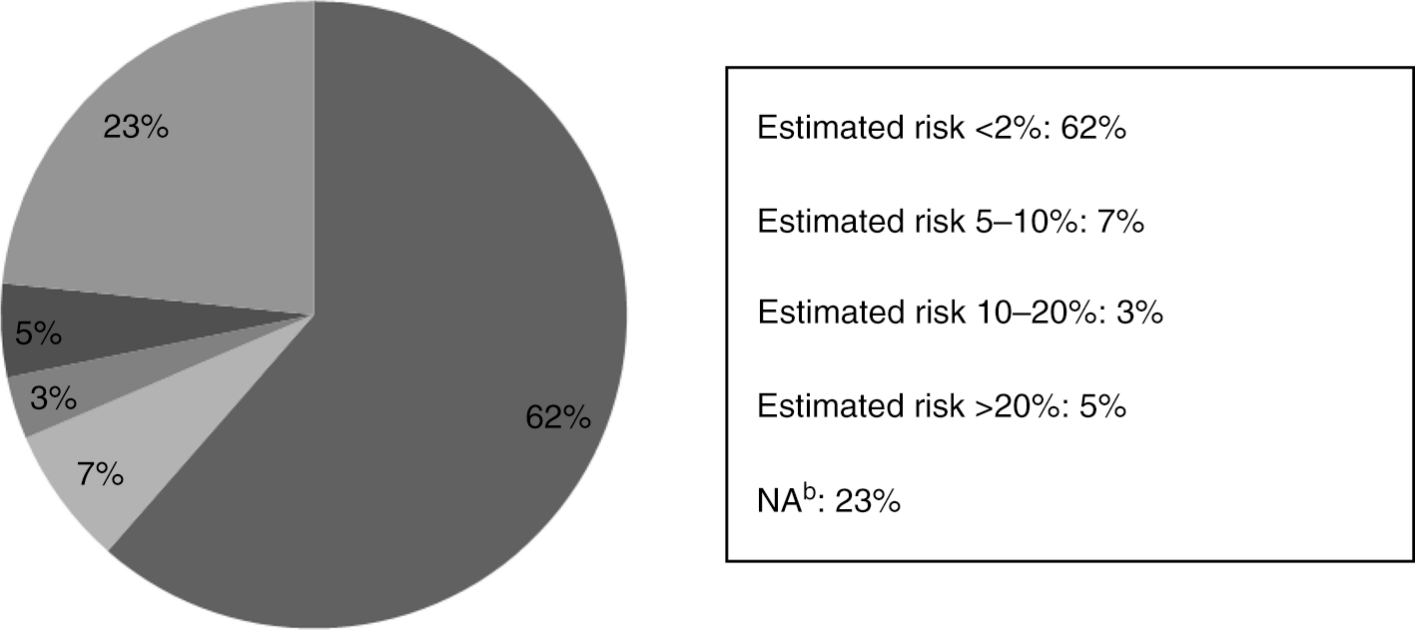
Risk of mother-to-child transmission, with all precautions taken, as estimated by women living with HIV.

A total of 344 (61%) women estimated the risk of MTCT, with all precautions taken, to be less than 2%; the remaining almost 40% either overestimated the risk or gave no answer (Figure 2). Women over 35 years of age overestimated the risk of MTCT, although women with a higher education had a tendency to answer correctly (Table 2).

## Discussion

This study is, to our knowledge, the largest survey on sexuality and fertility in a female HIV-positive population living in an industrialized setting. We found that the majority of women perceived mild or no symptoms of their chronic HIV infection. Most were sexually active and in a steady, HIV sero-discordant relationship, using condoms as the sole form of contraception. One-third of women in steady relationships were sexually inactive. This finding emphasizes the importance of speaking openly with patients about sexuality, because ignorance about HIV and risk of transmission may cause sexual abstinence. The majority of the women had children and one-quarter of the study participants desired pregnancy.

### Demographic characteristics

The large proportion of women experiencing mild or no symptoms of HIV correlates well with the high median CD4 cell counts and fully suppressed viral loads; this proportion is as high or even higher than several similar European studies conducted on WLWH [18,19]. A possible selection bias may be present, as women with few symptoms may have been asked to participate preferentially to women with severe symptoms. This is supported by the fact that there were significantly more women with a fully suppressed viral load and a high CD4 count among the women participating in the study than among the women who did not. However, there was no significant difference between the number of AIDS diagnoses in the two groups. Another possible bias was the significant difference in ethnicity among the two groups, with a larger number of white women of European descent among the study participants.

Most women of African origin in Denmark and Finland are of immigrant origin. There might be a possible answer bias linked to the different ethnicities, because the women of African origin may have experiences that differ from women of European origin.

### Sexuality

We found a majority of the study participants to be in a steady relationship, of which over half had an HIV-negative partner and two-thirds were sexually active. This finding is less than that presented in a national US cohort survey of sexual health among 3,111 HIV-negative women [20], which found that 96% of women aged 25 to 54 years who were living with a partner were sexually active within the last 12 months. It was recently observed that WLWH had lower sexual satisfaction compared to HIV-negative women. In addition, there seemed to be a change in sexual desire over time, with diminished sexual desire at the onset of ART compared to after a longer treatment duration [21]. Reasons for reduced desire were, among others, fear of transmission to an HIV-negative partner, issues related to disclosure, stigmatization, lack of energy and, in some countries, criminalization due to HIV [21].

In our study, almost 40% of the women did not use any kind of contraception; two-thirds of them were sexually active. One-quarter did not report the reason for not using contraceptives. This finding concurs with a Danish nationwide survey among PLWH, where 14% of heterosexual HIV-positive persons reported having sex without a condom during the previous 12 months and 15% did not answer the question [7]. A development towards condomless sex has been seen, possibly due to dramatically reduced transmission risks when viral load is fully suppressed [22]. The low transmission risks may alter views on sexuality and pregnancy desires and induce a need for contraception other than condoms among WLWH.

### Fertility

Most women in our survey reported previous pregnancies, and most had given birth prior to being diagnosed with HIV, including the majority of women with HIV-infected children. Of the women who reported having one or more HIV-infected children, one-third were pregnant at the time of HIV diagnosis. This finding stresses the importance of prenatal HIV screening: preventing MTCT is a feasible and realistic goal. In Finland, antenatal screening has been offered to pregnant women since 1998 [23]. In Helsinki, between 1993 and 2003, 18 of 45 pregnant HIV-positive women were diagnosed during pregnancy [23]. In Denmark, since January 1, 2010, all pregnant women have been offered screening for HIV [24]. In 2012, 34 HIV-positive pregnant women were identified, of whom five were unaware of their diagnosis [25]. The screening programs offer important possibilities for preventing MTCT.

Denmark is currently the only Nordic country offering fertility treatments for both parties, on terms similar to HIV-unaffected couples [26,27]. In Finland, fertility treatment is not available for either serodiscordant or seroconcordant couples [28], although possible amendments in legislation are underway, endorsing *in vitro* fertilization (IVF) treatments to HIV-affected couples (IA, personal communication).

A quarter of the WLWH in our survey had tried to conceive without success. In HIV-negative women, an estimated 50% attempting pregnancy succeed within three months [29]. According to the Danish Fertility Association, 4% of women aged 50 years report having attempted pregnancy, unsuccessfully, at some point in life [29]. A quarter of WLWH attempting pregnancy at the time of the study had been trying for more than 18 months. Although the Danish Fertility Association [29] recommend an examination for fertility when a couple has been unsuccessful at achieving pregnancy after one year, only half of these women had undergone a fertility examination. We found that women of African origin and women with lower CD4 counts experienced unsuccessful pregnancy attempts more often than women of white European or Asian origin and with higher CD4 counts.

Our findings are supported by Linas *et al*. [10], who showed in a study of over 900 enrolled patients that WLWH had a 40% reduction in the incidence of pregnancy, compared with HIV-negative women. Coll *et al*. [11] also found a lower incidence of achieved pregnancies among WLWH, who received IVF where the patient's own oocytes were used, relative to age-matched, HIV-negative women. In a Spanish study from 2007, the fertility of WLWH who sought preconceptive counselling was examined and almost 28% of the women undergoing a hysterosalpingography had tubal occlusion [30]. That is far more than the 14% previously reported in subfertile, HIV-negative women [31]. The authors found no explanation for the tubal anomalies, only suggesting that all WLWH seeking prenatal counselling should be offered a hysterosalpingography [30].

In our study, we found that a quarter of the women wished to become pregnant at the time of the study, and the good treatment possibilities had affected almost half of the women in a positive way. Women below the age of 35 were significantly more likely to indicate a desire for pregnancy than older women. In addition, interestingly, women with CD4 counts between 200 and 350 were more likely to indicate a desire for pregnancy than women with high CD4 counts.

A Swiss study among 114 HIV-positive individuals showed that 20% of the WLWH aged 20 to 40 years wanted to have children [32]. Similarly, a Canadian study of 230 WLWH found that a quarter of the women of fertile age had the intention to have children [33]. The authors found intentions to have children among WLWH approaching the levels of the general population [33].

Attempts have been made, in United Kingdom and North America, to examine the desire for children and the wish for fertility treatments among PLWH [14,15] [34]. These studies suggest that WLWH have a strong desire for children, despite their HIV diagnosis. Fiore *et al*. [18] investigated the reproductive health characteristics of WLWH living in Europe (Spain, Italy, Ukraine, France and Poland) in 2003 and 2004, through a cross-sectional questionnaire, and the results suggested that knowledge of HIV infection neither influences the desire for children nor the decisions regarding pregnancy. However, a multicentre questionnaire study conducted in Italy in 2010 and 2011 on WLWH of childbearing age found that 61% of the women did not want to become pregnant [13]. The HIV diagnosis had affected half of the women negatively in relation to their desire for pregnancy [13].

Although the results of our survey suggest that a greater number of the WLWH in the two Nordic countries have a desire for children, it is important to highlight the fact that almost one-sixth of the study participants reported that the HIV diagnosis ended that desire.

### Mother-to-child transmission

Though the majority of the women correctly estimated the MTCT risk at lower than 2% [35–37], one can only speculate why as many as one-quarter chose not to answer the question. An alarming reason might be misconception of the risk, as 15% of the women responding to this question overestimated the risk. The median age of the women estimating the risk of MTCT to be above 2% was 47 years. It is indeed worrisome that possibly a large number of women do not know the true risks of MTCT and may potentially make life-changing decisions, such as not having children, due to overestimation of the risk. However, information concerning MTCT might not be a priority for women who do not wish to become pregnant, already have the number of children they want or have reached an age when pregnancy no longer is possible. This theory is supported by the significantly higher percentage of older women overestimating the risk of MTCT, although women with higher education were more likely to estimate the risk correctly.

Ammassari *et al*. [13] found a much larger proportion, one-fifth of the participating Italian WLWH, reporting the risk of MTCT, all precautions taken, to be 50%.

In our study, the response rates varied across the questionnaire and there was a tendency for lower response rates to more sensitive questions. This phenomenon might indicate self-stigmatization among the study participants, despite the anonymous nature of the survey, and may also be considered as a limitation to the study.

The study strengths are the large number of study participants, as well as the fact that the study was conducted nationwide in Denmark. Together with the largest Department of Infectious Diseases in Finland, we recruited from clinics covering >90% and >50% of the female HIV-positive population in Denmark and Finland, respectively. The questionnaire was developed after research in the field, resulting in questions that were varied and precise and explored a wide range of aspects concerning sexuality and fertility.

A major weakness was the fact that not all women attending one of the seven clinics were included, mainly due to language or psychosocial barriers. In addition, the number of premenopausal women answering the questions concerning menopause made this section not suitable for analysis.

## Conclusions

In conclusion, the majority of WLWH in an industrialized setting in Denmark and Finland have few HIV-related symptoms, are sexually active and have a strong desire for children. We believe that the findings in our study emphasize the importance of health personnel asking WLWH about their partners, contraception and fertility wishes, at least annually at routine check-ups. There is no possibility of discovering patients’ problems and concerns and ultimately finding solutions unless these issues are addressed in the open.

## Supplementary Material

Perception of sexuality and fertility in women living with HIV: a questionnaire study from two Nordic countries
