# Supplementary material for: Perception of sexuality and fertility in women living with HIV: a questionnaire study from two Nordic countries
Source: J Int AIDS Soc. 2015 Jun 1;18(1):19962. doi: 10.7448/IAS.18.1.19962 (PMC4452736; doi:10.7448/IAS.18.1.19962)
Supplement: Perception of sexuality and fertility in women living with HIV: a questionnaire study from two Nordic countries [file JIAS-18-19962-s001.pdf]

Questionnaire regarding pregnancy, fertility and menopause

**Patient label**

**Date:**

1. Marital status
- Married .... ☐
- Cohabiting .... ☐
- Steady relationship .... ☐
- Divorced (continue to question 3.) .... ☐
- Widow (continue to question 3.) .... ☐
- Single (continue to question 3.) .... ☐
- Do not wish to answer (continue to question 3.) .... ☐

2. Partner's HIV status?
- HIV positive .... ☐
- HIV negative .... ☐
- Partner has not undergone test .... ☐
- Do not know .... ☐
- Do not wish to answer .... ☐

3. How will you describe your present symptoms of HIV?
- None .... ☐
- Mild .... ☐
- Moderate .... ☐
- Severe .... ☐
- Do not wish to answer .... ☐

4. Do you smoke?
- Yes .... ☐
- Ex-smoker (continue to question 5.) .... ☐
- No (continue to question 7.) .... ☐
- Do not wish to answer (continue to question 7.) .... ☐

5. How many cigarettes do you smoke/have you been smoking daily?
- __________cigarettes
- Do not wish to answer .... ☐

6. For how many years have you been smoking?
- __________years
- Do not wish to answer .... ☐

7. Education
- Elementary school .... ☐
- Grammar-school level or higher education .... ☐
- Do not wish to answer .... ☐

Questions regarding contraception and sexual activity

8. Are you sexually active?
- Yes ☐
- No (continue to question 10.) ☐
- Do not wish to answer (continue to question 10.) ☐

9. When did you last have sex?
- Within the last week ☐
- Within the last month ☐
- Within the last 6 months ☐
- Do not wish to answer ☐

10. Do you use contraception?
- Yes (continue to question 11.) ☐
- No (continue to question 12.) ☐
- Do not wish to answer (continue to question 13.) ☐

11. If yes, what kind (only cross one)?
- Condom ☐
- Condom + hormonal contraception (the pill/contraceptive implant) ☐
- Condom + IUD (hormone coil or copper coil) ☐
- Condom + sterilization ☐
- Hormonal contraception (the pill/contraceptive implant) ☐
- IUD (hormone coil or copper coil) ☐
- Sterilization ☐
- Other ☐
- Do not wish to answer ☐

12. If you do not use contraception - why do you not use regular contraception?
- Own decision ☐
- Partner's decision ☐
- Joint decision ☐
- Because my partner has HIV ☐
- Because I try to become pregnant ☐
- Because my viral load is low and the risk of infecting my partner is small ☐
- Because I am not sexually active ☐
- Do not wish to answer ☐

13. Were you sterilized after you were diagnosed with HIV?
- Yes ☐
- No (continue to question 15.) ☐
- Do not wish to answer (continue to question 15.) ☐

14. Subsequently, did you regret having been sterilized?
- Yes ☐
- No ☐
- No, I already had the number of children that I wanted at that point in time ☐
- Do not wish to answer ☐

Questions regarding pregnancy

15. Are you or have you ever been pregnant?

- Yes, previously .... ☐
- Yes, I am currently pregnant .... ☐
- No (continue to question 24.) .... ☐
- Do not wish to answer (continue to question 24.) .... ☐

16. Do you have children?

- Yes .... ☐
- No (continue to question 21.) .... ☐
- Do not wish to answer (continue to question 21.) .... ☐

17. If yes: please, write the year of your child's/children's birth (if twins, please write the same year twice):

- _____
- _____
- _____
- _____

18. If you have children, is one or more HIV-positive?

- Yes, how many: _____ .... ☐
- No .... ☐
- Do not wish to answer .... ☐

19. Do you live together with your children?

- Yes .... ☐
- No .... ☐
- Do not wish to answer .... ☐

20. If you have been pregnant, when did you become pregnant?

For child 1:

- Had child 1 <u>before</u> HIV diagnosis .... ☐
- Had child 1 <u>after</u> HIV diagnosis .... ☐
- Do not wish to answer .... ☐

How did you become pregnant?

- Unprotected sex .... ☐
- Unprotected sex after ovulation test .... ☐
- Self-insemination/self-fertilization .... ☐
- IVF (In Vitro Fertilization)/test tube fertilization .... ☐
- ICSI (Intra Cytoplasmatic Sperm Injection/Microinsemination .... ☐
- Do not know the name of the method .... ☐
- Other .... ☐
- Do not wish to answer .... ☐

For child 2:

- Had child 2 <u>before</u> HIV diagnosis .... ☐

- Had child 2 after HIV diagnosis .... ☐
- Do not wish to answer .... ☐

How did you become pregnant?
- Unprotected sex.. ☐
- Unprotected sex after ovulation test.. ☐
- Self-insemination/self-fertilization .... ☐
- IVF (In Vitro Fertilization)/test tube fertilization .... ☐
- ICSI (Intra Cytoplasmatic Sperm Injection/Microinsemination .... ☐
- Do not know the name of the method .... ☐
- Other .... ☐
- Do not wish to answer .... ☐

For child 3:
- Had child 3 before HIV diagnosis .... ☐
- Had child 3 after HIV diagnosis .... ☐
- Do not wish to answer .... ☐

How did you become pregnant?
- Unprotected sex.. ☐
- Unprotected sex after ovulation test.. ☐
- Self-insemination/self-fertilization .... ☐
- IVF (In Vitro Fertilization)/test tube fertilization .... ☐
- ICSI (Intra Cytoplasmatic Sperm Injection/Microinsemination .... ☐
- Do not know the name of the method .... ☐
- Other .... ☐
- Do not wish to answer .... ☐

For child 4:
- Had child 4 before HIV diagnosis .... ☐
- Had child 4 after HIV diagnosis .... ☐
- Do not wish to answer .... ☐

How did you become pregnant?
- Unprotected sex.. ☐
- Unprotected sex after ovulation test.. ☐
- Self-insemination/self-fertilization .... ☐
- IVF (In Vitro Fertilization)/test tube fertilization .... ☐
- ICSI (Intra Cytoplasmatic Sperm Injection/Microinsemination .... ☐
- Do not know the name of the method .... ☐
- Other .... ☐
- Do not wish to answer .... ☐

21. After being diagnosed with HIV did you experience:
- Spontaneous abortion.. ☐
- Had provoked abortion .... ☐
- Gave birth to a stillborn child... ☐
- Gave birth to living child/children.. ☐
- None of the above... ☐

- Do not wish to answer ☐

22. Were you pregnant when you were diagnosed with HIV?

- Yes ☐
- No (continue to question 24.) ☐
- Do not wish to answer (continue to question 24.) ☐

23. If yes did the pregnancy result in:

- Living child/children ☐
- Stillborn child/children ☐
- Spontaneous abortion ☐
- Provoked abortion ☐
- Do not wish to answer ☐

24. Currently, do you wish to become pregnant?

- Yes ☐
- No ☐
- I have the number of children that I want ☐
- Do not wish to answer ☐

25. When you were diagnosed with HIV did this change your opinion about how many children you wanted to have and at what point in time?

- I wanted to have children earlier ☐
- It had no importance for my wish ☐
- I already had the number of children that I wanted ☐
- I never wanted to have children ☐
- I wanted to have children later on ☐
- The diagnosis caused that I did no longer want to have children ☐
- Other ☐
- Do not wish to answer ☐

26. Have the good treatment possibilities for HIV influenced your wish to have children?

- Yes ☐
- No ☐
- I never wanted to have children ☐
- Do not know ☐
- Do not wish to answer ☐

Questions regarding fertility

27. Age when you had your first period?
- years
- Do not wish to answer ☐

28. Did you try to become pregnant without having success?
- Yes ☐
- No ☐
- Do not wish to answer ☐

29. Currently, do you try to become pregnant?
- Yes ☐
- No (continue to question 31.) ☐
- Do not wish to answer (continue to question 31.) ☐

30. For how long have you tried to become pregnant?
- <6 months ☐
- 6–18 months ☐
- >18 months ☐
- Do not wish to answer ☐

31. Have you ever been examined regarding your ability to become pregnant?
- Yes ☐
- No (continue to question 33.) ☐
- Do not wish to answer (continue to question 33.) ☐

32. If yes: Where did the examination take place?
- Hospital ☐
- General practitioner ☐
- Private gynecologist ☐
- Abroad ☐
- Other ☐
- Do not wish to answer ☐

33. Did you have regular periods during the last 12 months?
- Yes (continue to question 40.) ☐
- No ☐
- Irregular periods but probably due to other cause than menopause (continue to question 40.) ☐
- Do not wish to answer ☐

34. Did you have regular periods during the last 3-12 months but not during the last 2 months?
- Yes (continue to question 36.) ☐
- No ☐

Do not wish to answer ☐

35. Did you have your period during the last 12 months?

- Yes (continue to question 38.) ☐
- No ☐
- Do not wish to answer (continue to question 38.) ☐

36. Why did you stop having your period?

- It happened naturally ☐
- It was caused by an operation where I had my ovaries and/or uterus removed ☐
- It happened as a result of chemotherapy and/or radiation therapy for cancer ☐
- I have stopped having my period due to hormonal treatment, contraceptive implant or the like ☐
- Other reason ☐
- Do not wish to answer ☐

37. Age when your periods stopped?

- _______ years
- Do not wish to answer ☐

38. Do you have symptoms of beginning menopause?
You should only answer this question if your periods are irregular or have stopped and if you are younger than 55 years (more answers possible)

- Hot flushes ☐
- Night sweats ☐
- Sleep disturbances ☐
- Heart throbs/palpitations ☐
- Chest pain/weight on the chest ☐
- Shortness of breath ☐
- Changed lust for sex ☐
  If yes: increased lust ☐
  decreased lust ☐
- Pain during intercourse ☐
- Dry mucous membrane of the vagina ☐
- Concentration difficulty ☐
- Loss of memory ☐
- Impatience/nervousness ☐
- Depression ☐
- Anxiety ☐
- General emotional problems ☐
- Tiredness/fatigue ☐
- Headache ☐
- Numbness ☐
- Gastrointestinal symptoms ☐
- Dizziness ☐
- Pain in the joints ☐
- Increased weight ☐
- Loss of bladder control ☐
- Do not wish to answer ☐

39. Do you get hormonal treatment for symptoms of the climacteric?

- Yes .... ☐
- No .... ☐
- Do not wish to answer .... ☐

40. If all measures to reduce the risk for HIV infection from mother to child are met (mothers HIV RNA immeasurable, the child gets HIV medicine for 4 weeks after birth and breast feeding is omitted) how big do you think that the risk of infection from mother to child is?

- <2% .... ☐
- 5-10% .... ☐
- 10-20% .... ☐
- >20% .... ☐
- Do not wish to answer .... ☐
